# Supplementary material for: Using Flavonoid Substitution Status to Predict Anticancer Effects in Human Melanoma Cancers: An In Vitro Study
Source: Cancers (Basel). 2024 Jan 23;16(3):487. doi: 10.3390/cancers16030487 (PMC10854695; doi:10.3390/cancers16030487)
Supplement: Supplementary file 1 [file cancers-16-00487-s001.zip › cancers-2795780-supplementary.pdf]

Supplementary Materials

A375 24 h

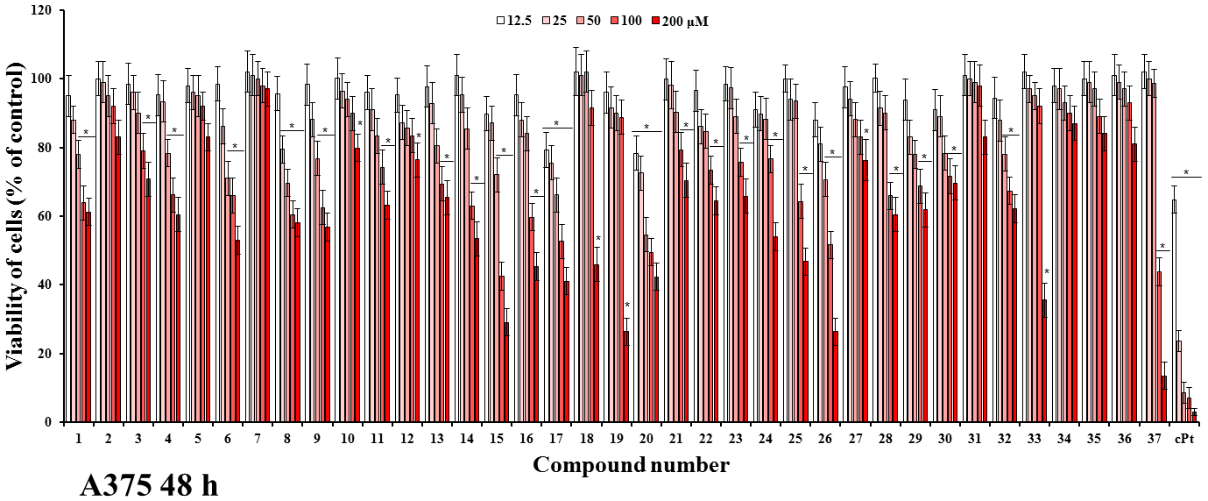

A375 48 h

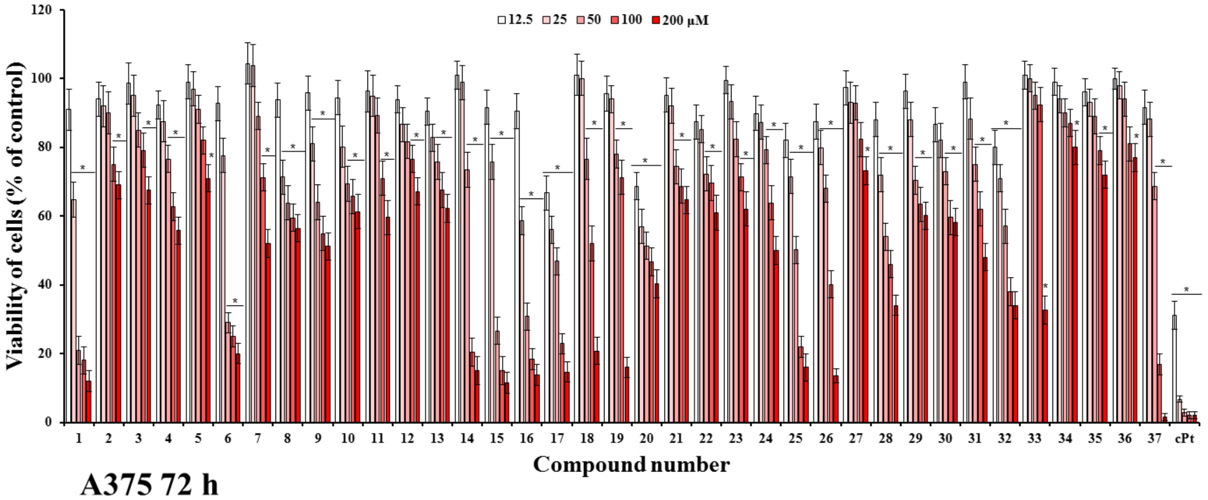

A375 72 h

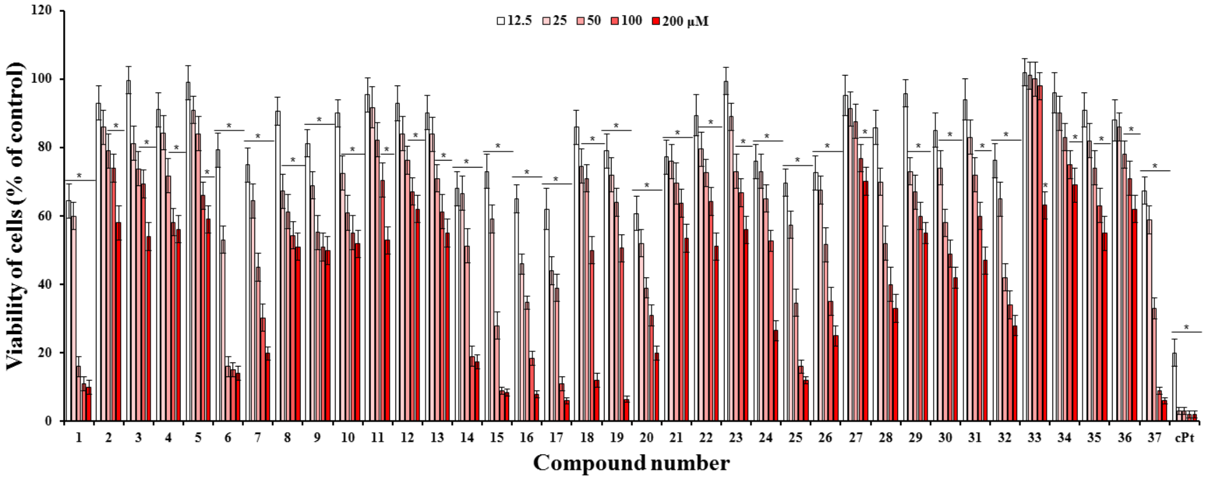

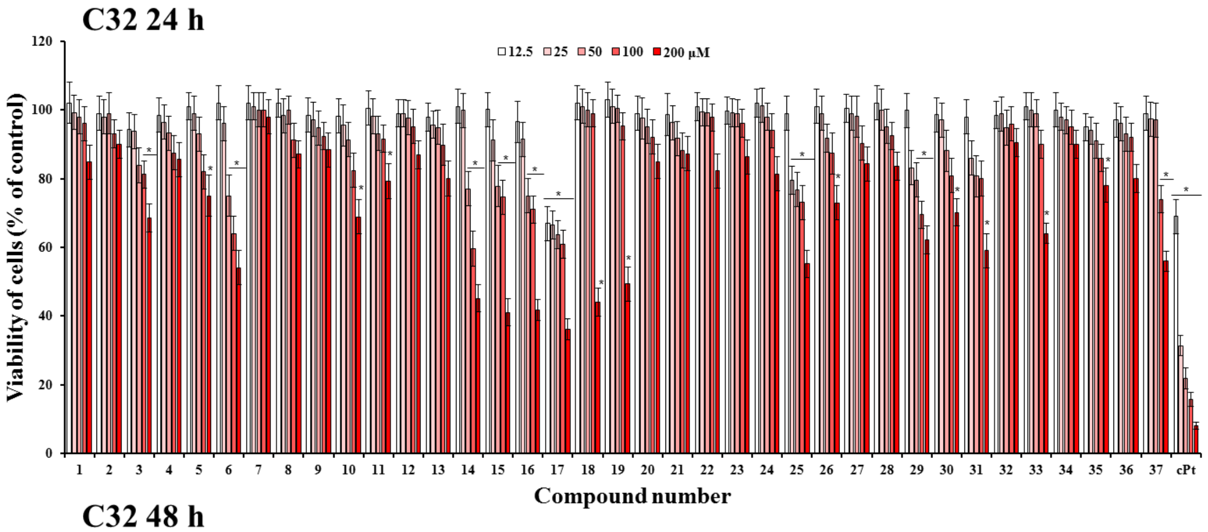
C32 24 h
12.5 25 50 100 200 µM
Viability of cells (% of control)
Compound number

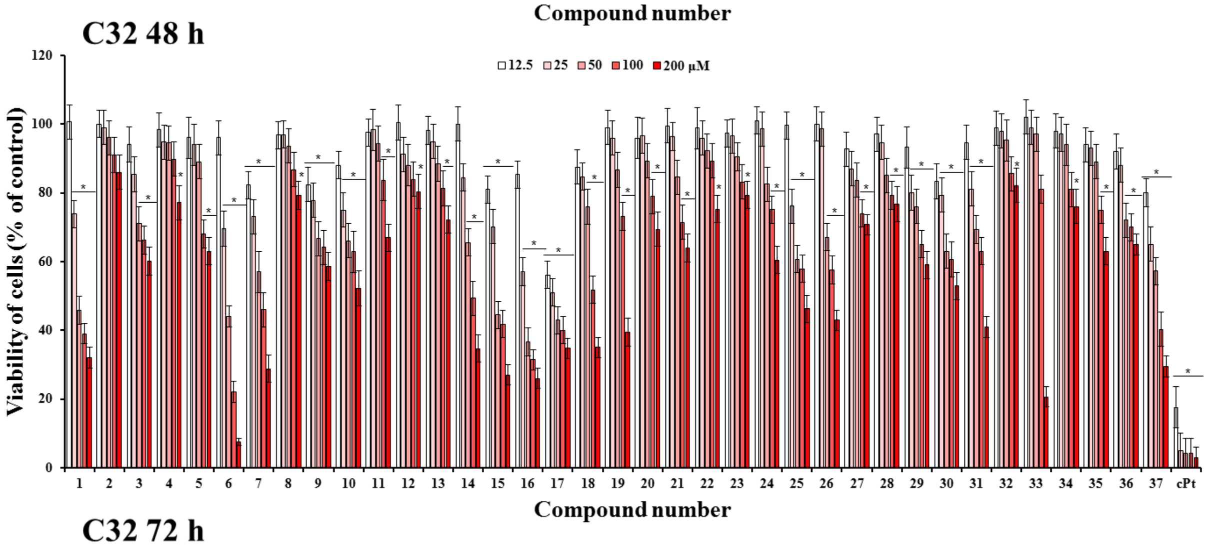
C32 48 h
12.5 25 50 100 200 µM
Viability of cells (% of control)
Compound number

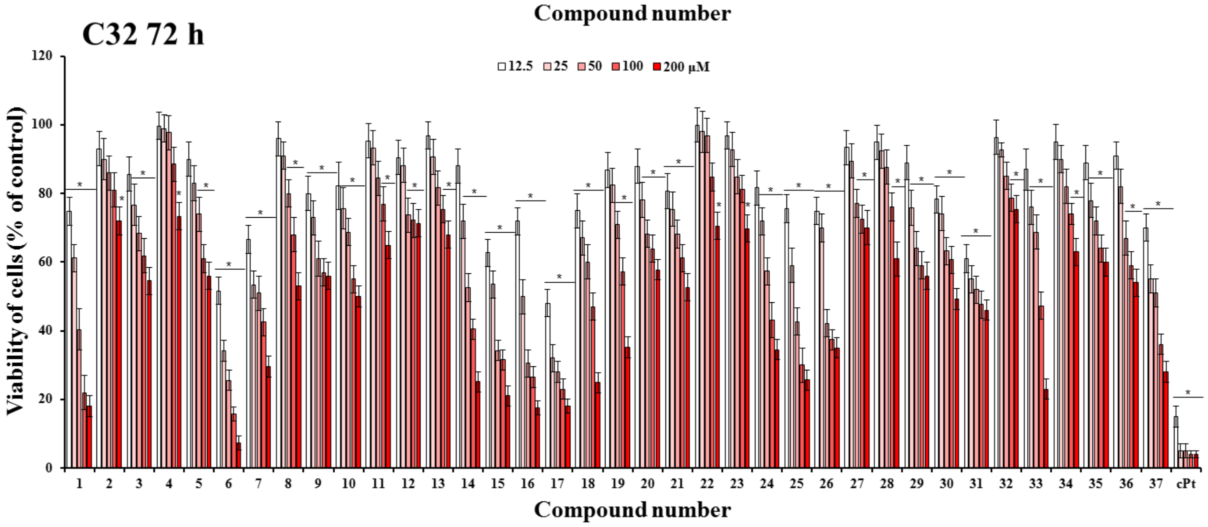
C32 72 h
12.5 25 50 100 200 µM
Viability of cells (% of control)
Compound number

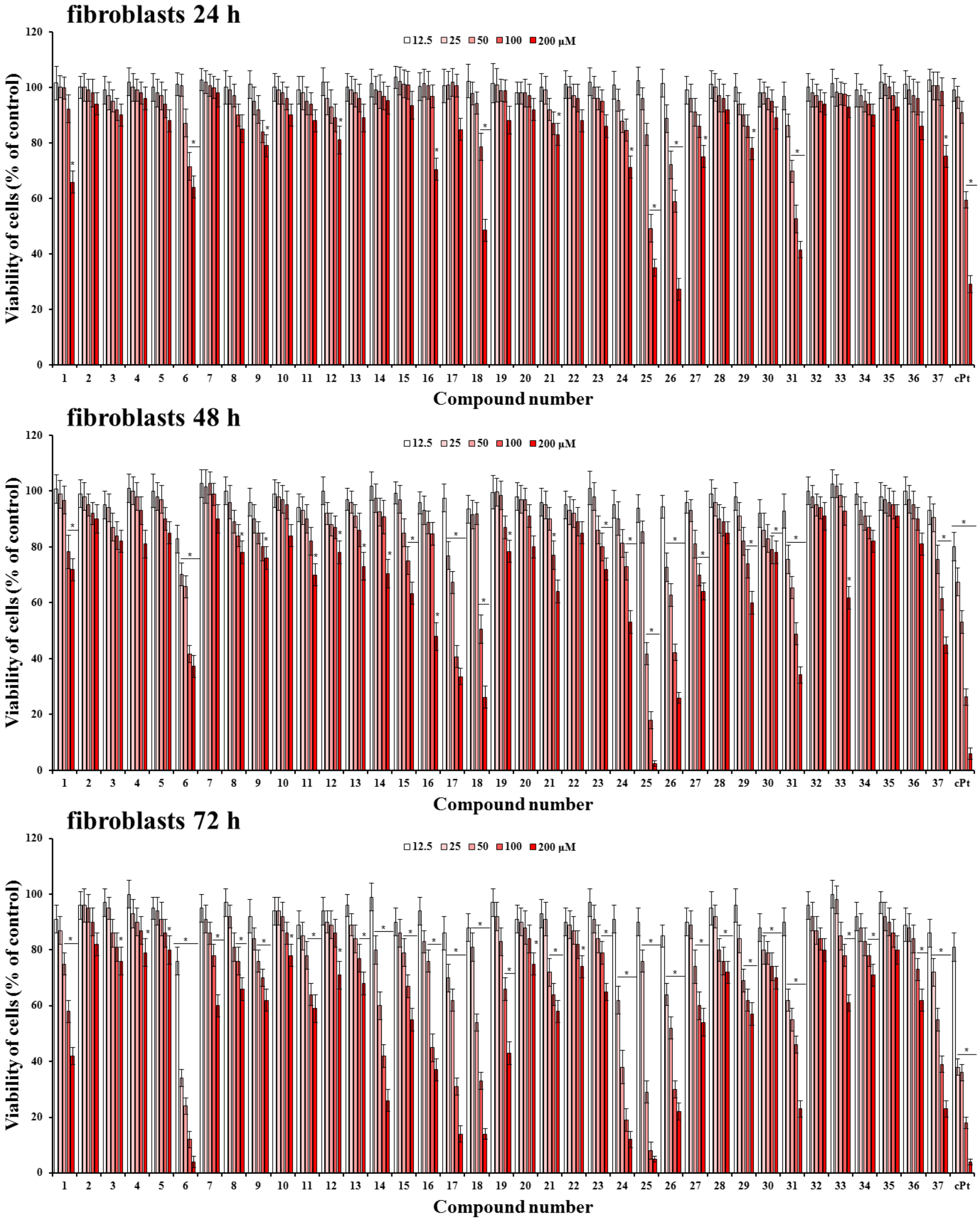

Figure S1. Viability of A375, C32 melanoma cells and dermal fibroblasts treated with compounds 1–37 or cisplatin for 24, 48 and 72 hours determined by a MTT assay. Data are presented as mean ± SD from three independent experiments. * $p < 0.05$ compared to control group.

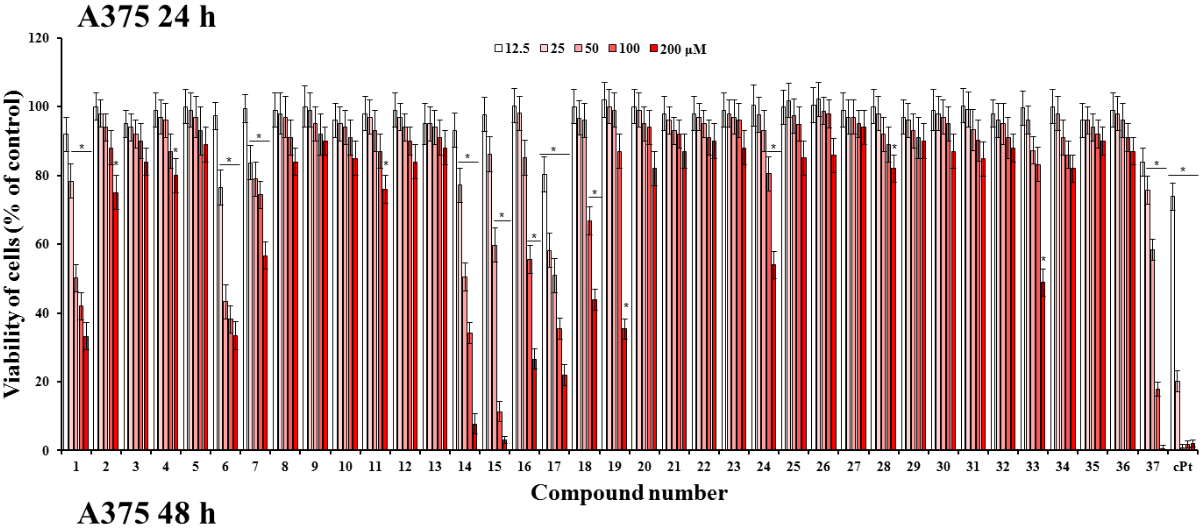
A375 24 h
12.5 25 50 100 200 µM
Viability of cells (% of control)
0 20 40 60 80 100 120
1 2 3 4 5 6 7 8 9 10 11 12 13 14 15 16 17 18 19 20 21 22 23 24 25 26 27 28 29 30 31 32 33 34 35 36 37 cPt
Compound number

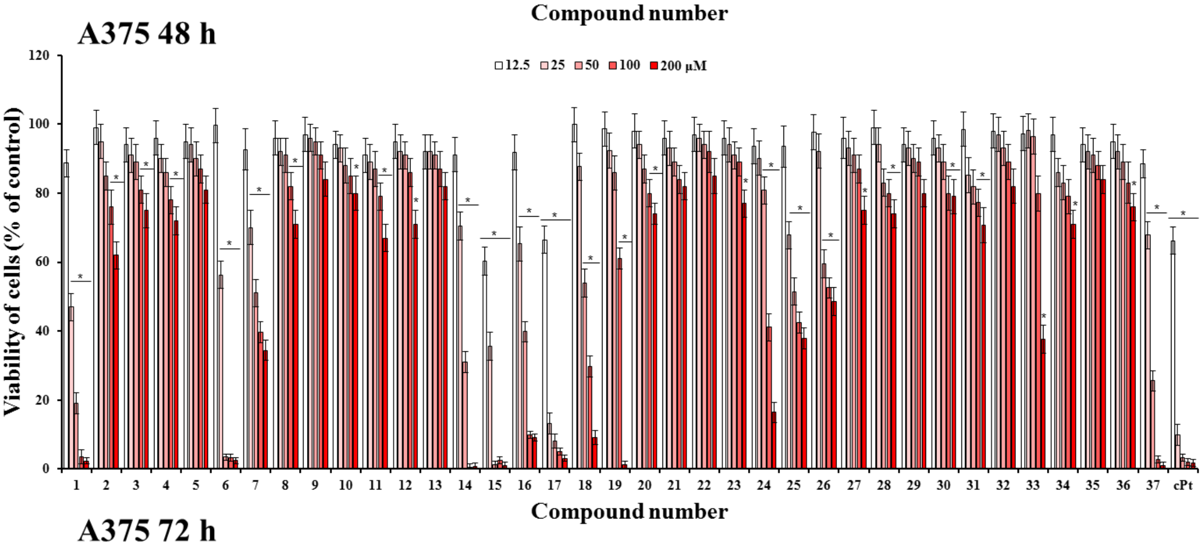
A375 48 h
12.5 25 50 100 200 µM
Viability of cells (% of control)
0 20 40 60 80 100 120
1 2 3 4 5 6 7 8 9 10 11 12 13 14 15 16 17 18 19 20 21 22 23 24 25 26 27 28 29 30 31 32 33 34 35 36 37 cPt
Compound number

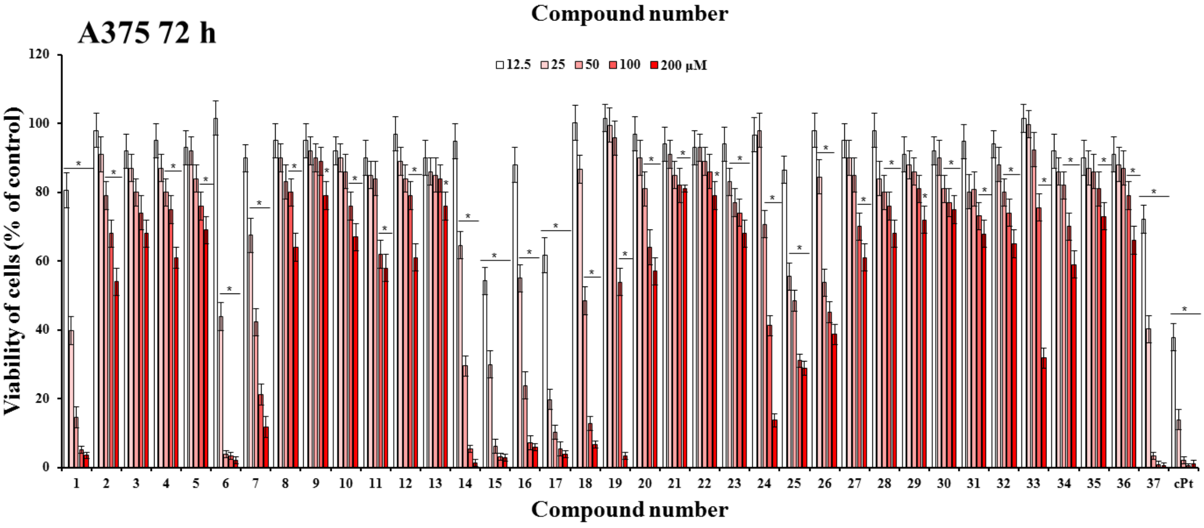
A375 72 h
12.5 25 50 100 200 µM
Viability of cells (% of control)
0 20 40 60 80 100 120
1 2 3 4 5 6 7 8 9 10 11 12 13 14 15 16 17 18 19 20 21 22 23 24 25 26 27 28 29 30 31 32 33 34 35 36 37 cPt
Compound number

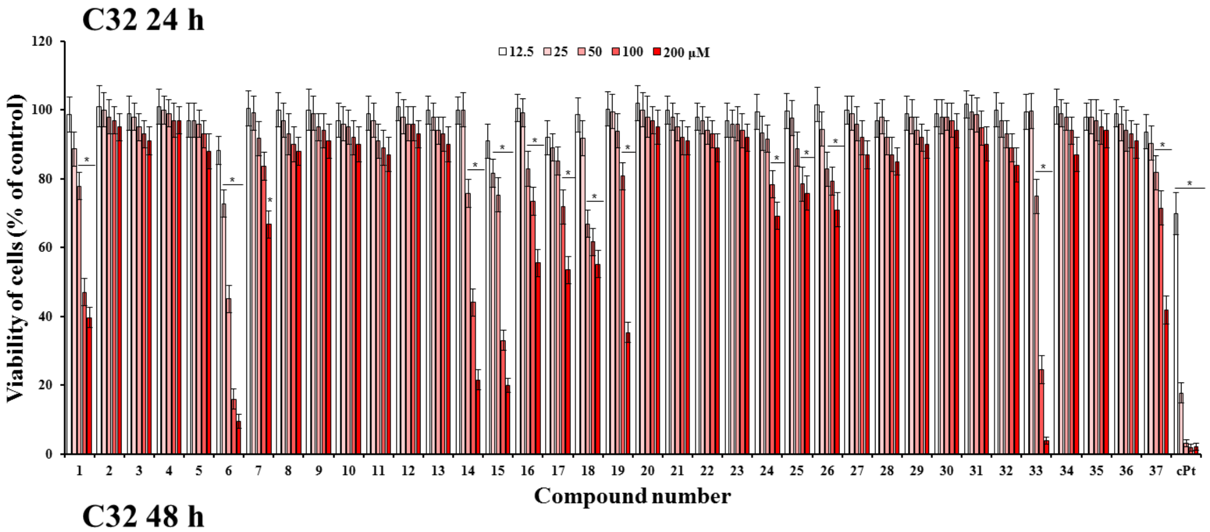
C32 24 h
12.5 25 50 100 200 µM
Viability of cells (% of control)
Compound number
cPt

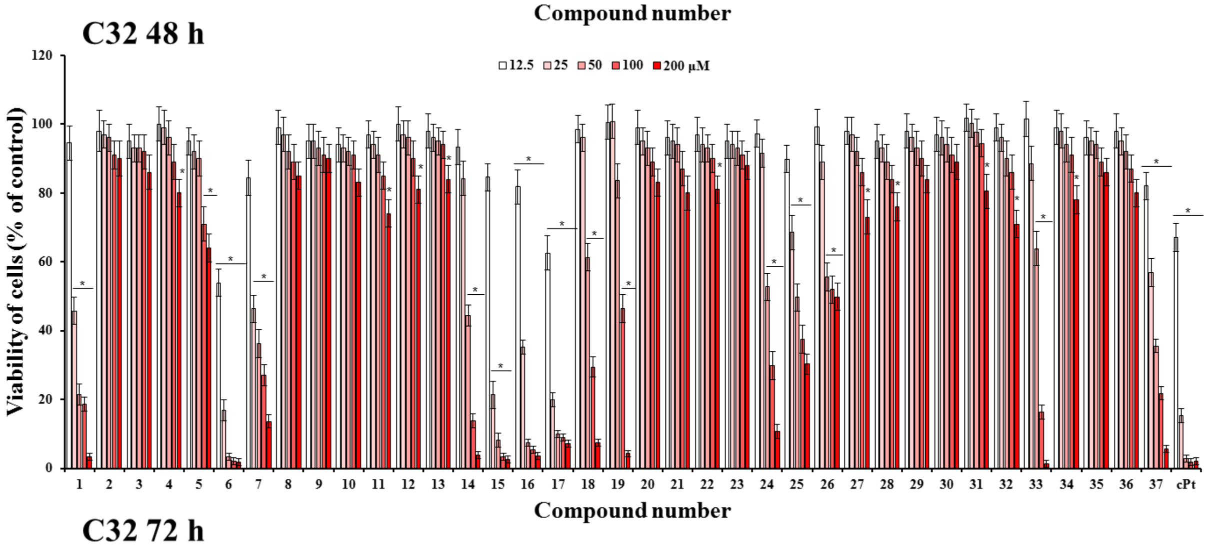
C32 48 h
12.5 25 50 100 200 µM
Viability of cells (% of control)
Compound number
cPt

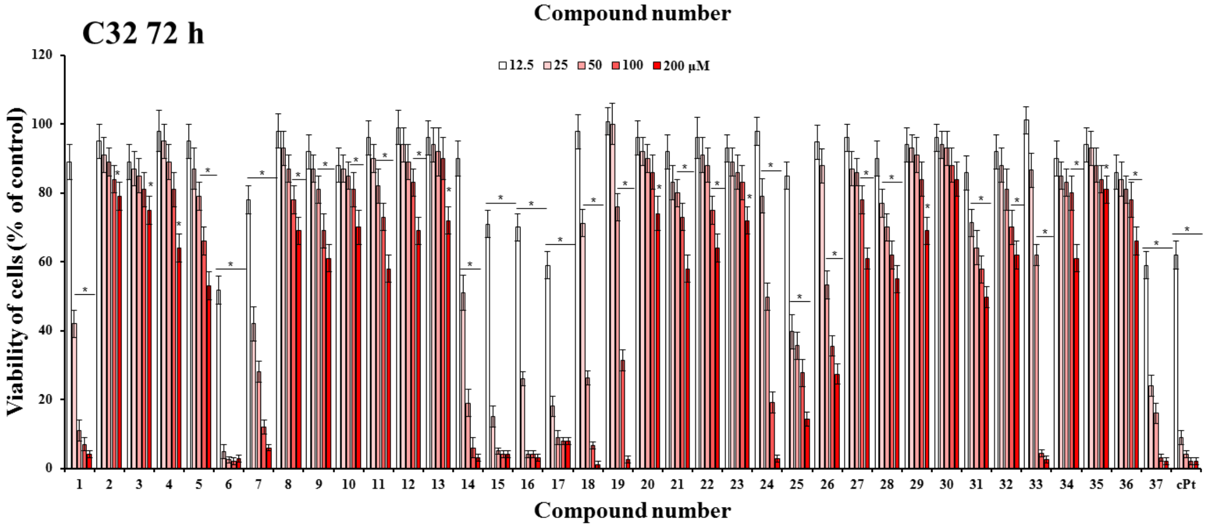
C32 72 h
12.5 25 50 100 200 µM
Viability of cells (% of control)
Compound number
cPt

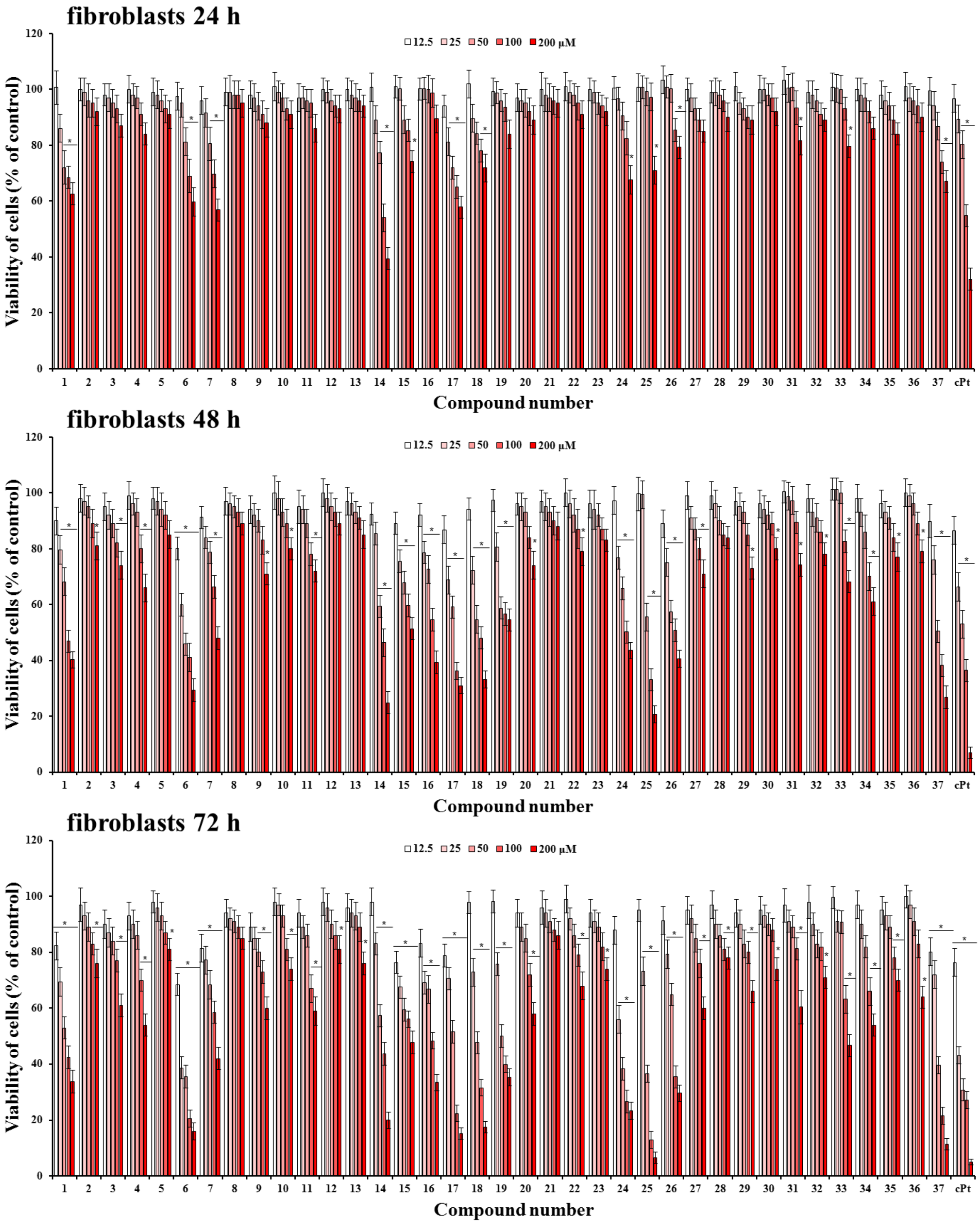

Figure S2. Viability of A375, C32 melanoma cells and dermal fibroblasts treated with compounds 1–37 or cisplatin for 24, 48 and 72 hours determined by a neutral red uptake assay. Data are presented as mean ± SD from three independent experiments. * $p < 0.05$ compared to control group.

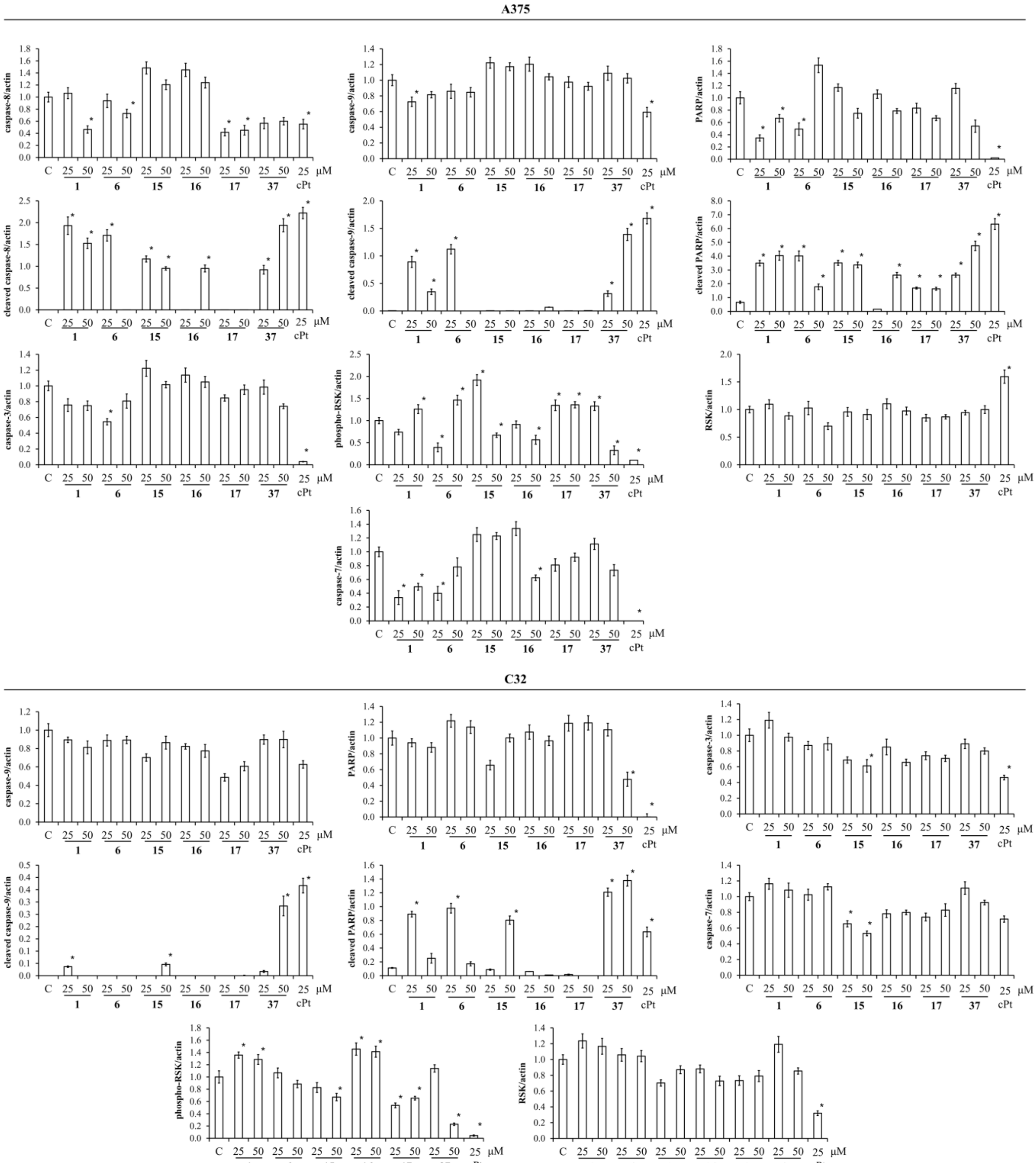

Figure S3. Quantitative analysis of western immunoblot images. Data are presented as mean ± SD. * $p < 0.05$ compared to control group.

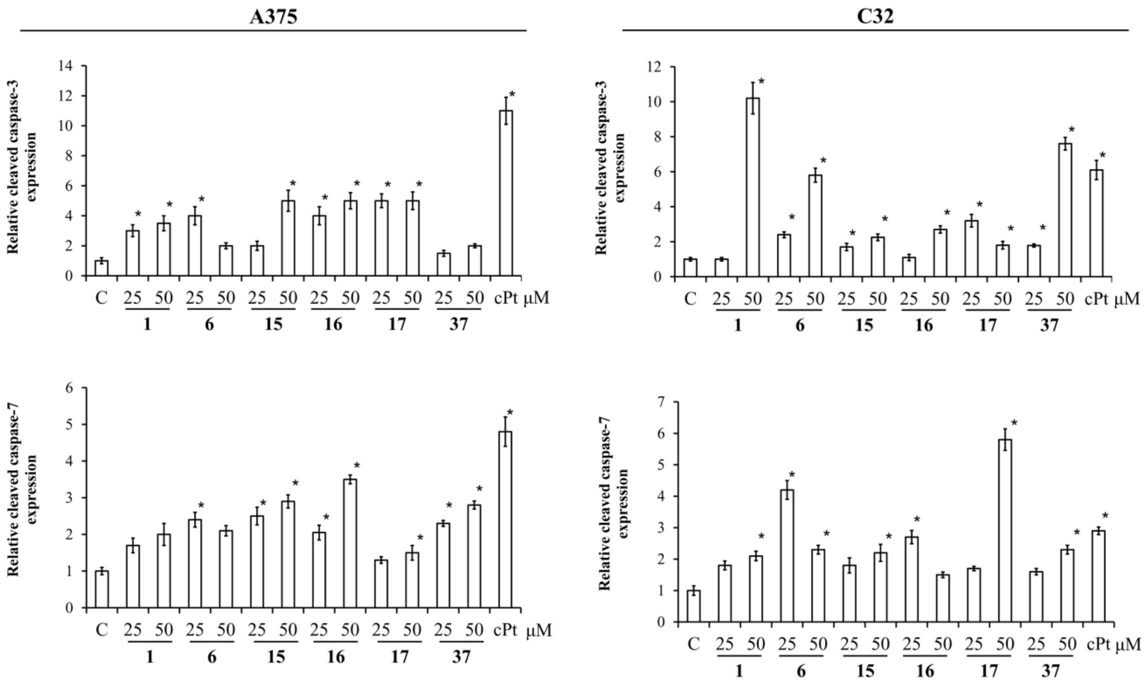

Figure S4. Quantitative analysis of immunofluorescence images. Data are presented as mean ± SD. * $p < 0.05$ compared to control group.

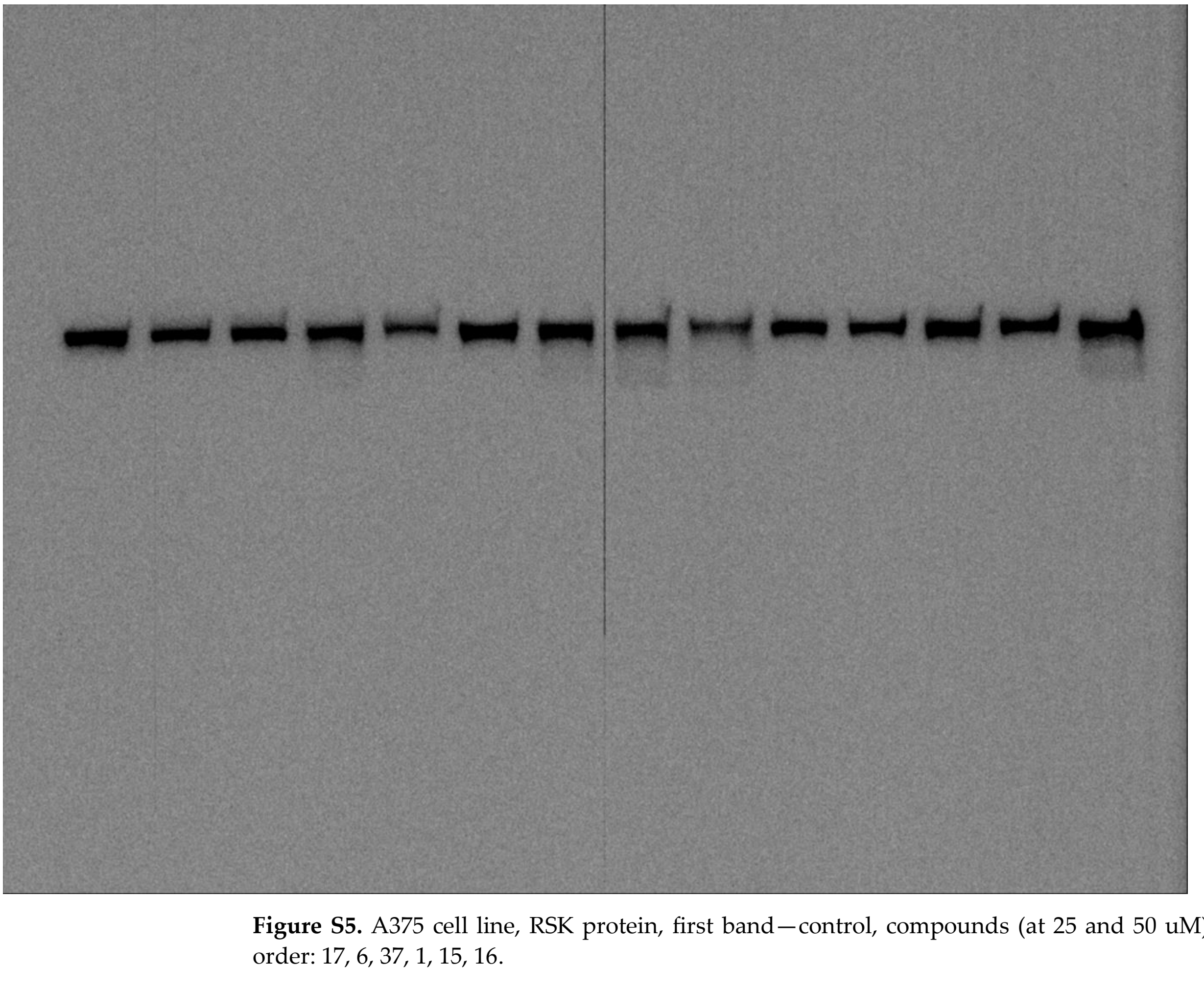

Figure S5. A375 cell line, RSK protein, first band—control, compounds (at 25 and 50 uM) in the order: 17, 6, 37, 1, 15, 16.

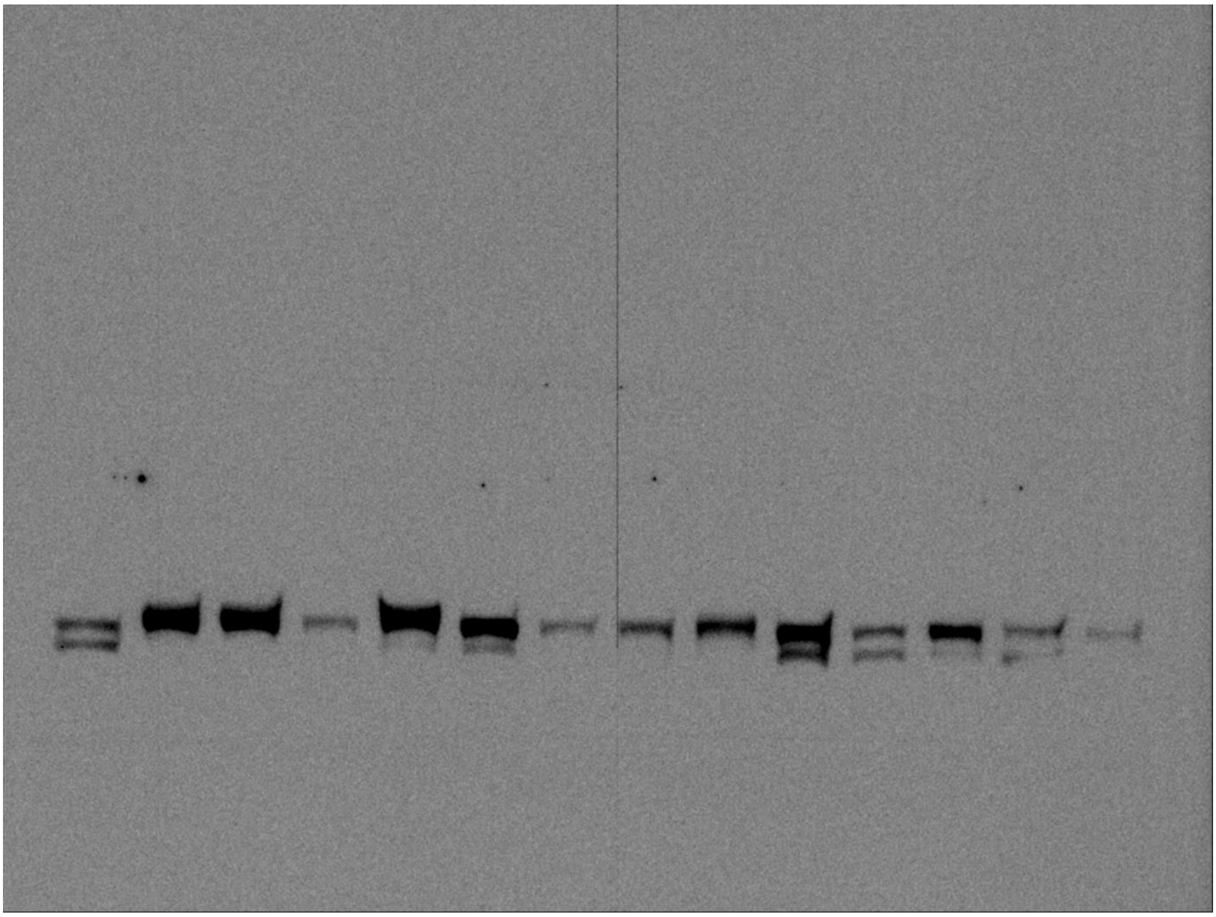

Figure S6. A375 cell line, pRSK protein, first band—control, compounds (at 25 and 50 uM) in the order: 17, 6, 37, 1, 15, 16.

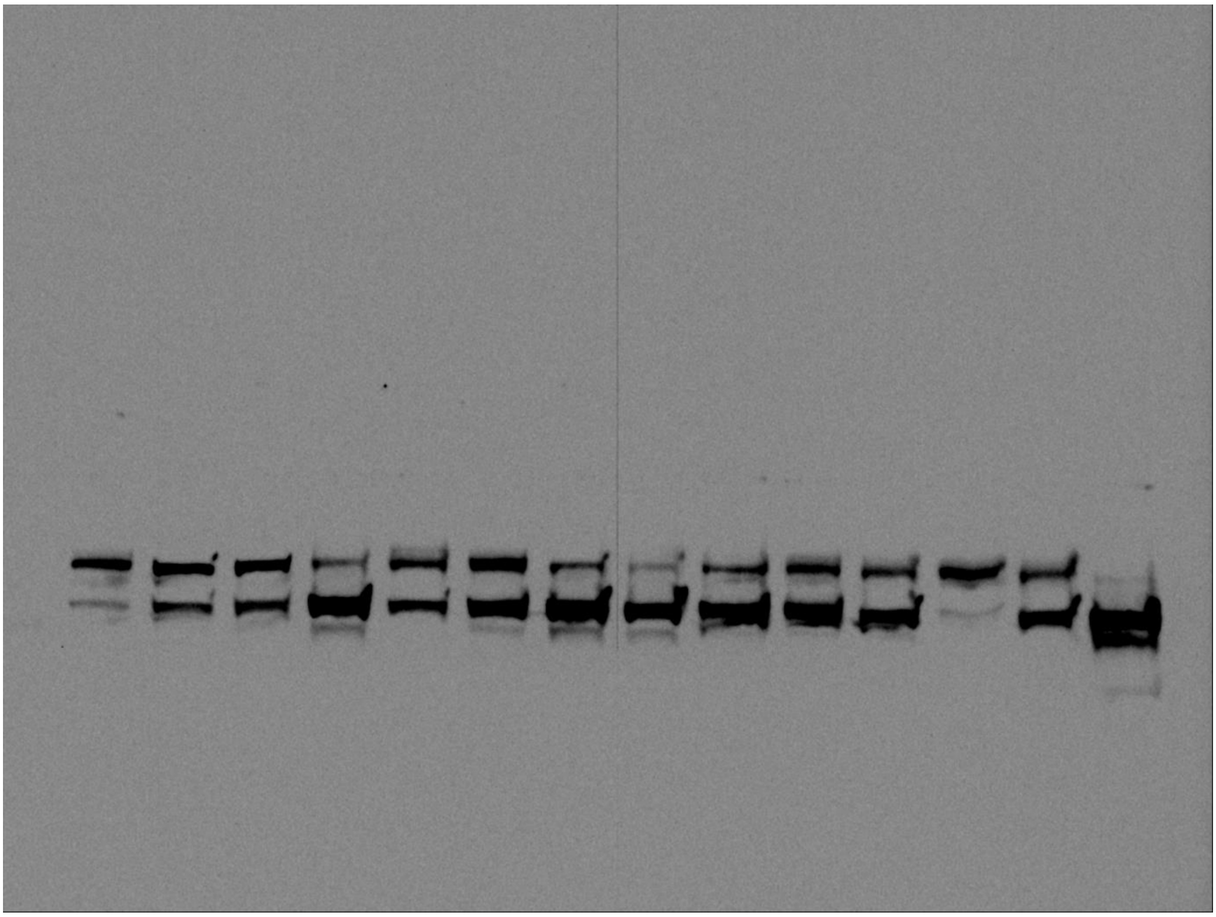

Figure S7. A375 cell line, PARP and cPARP, first band—control, compounds (at 25 and 50 uM) in the order: 17, 6, 37, 1, 15, 16.

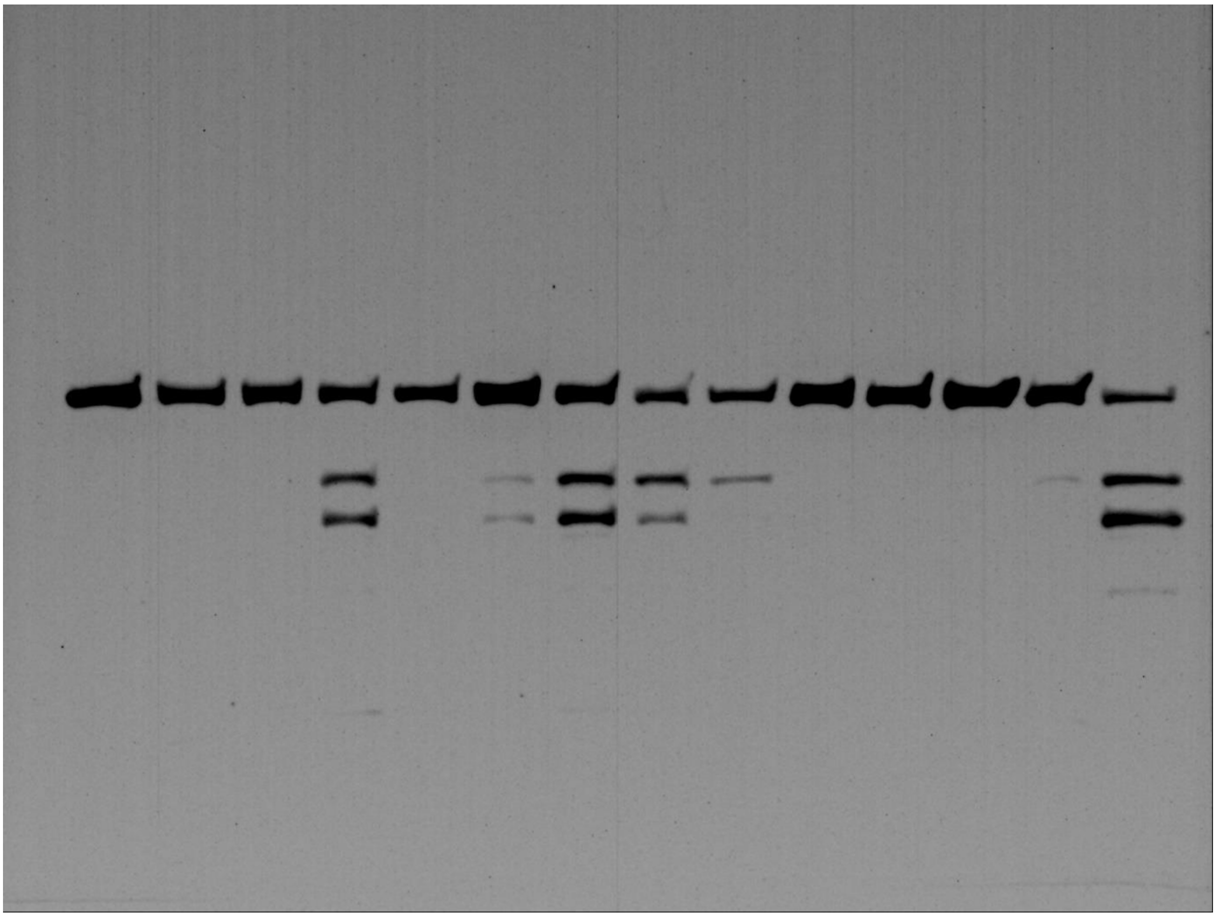

Figure S8. A375 cell line, caspase 9, first band—control, compounds (at 25 and 50 uM) in the order: 17, 6, 37, 1, 15, 16.

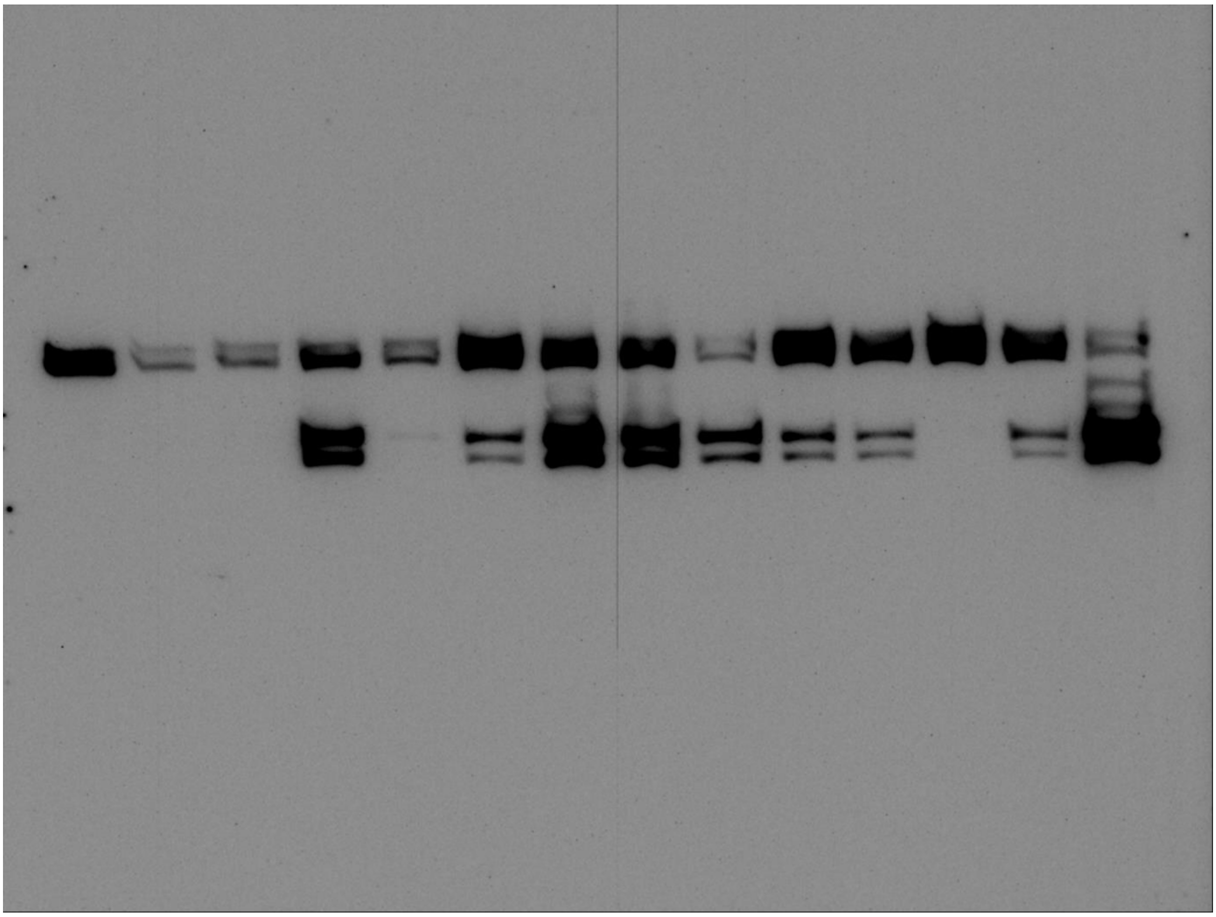

Figure S9. A375 cell line, caspase 8, first band—control, compounds (at 25 and 50 uM) in the order: 17, 6, 37, 1, 15, 16.

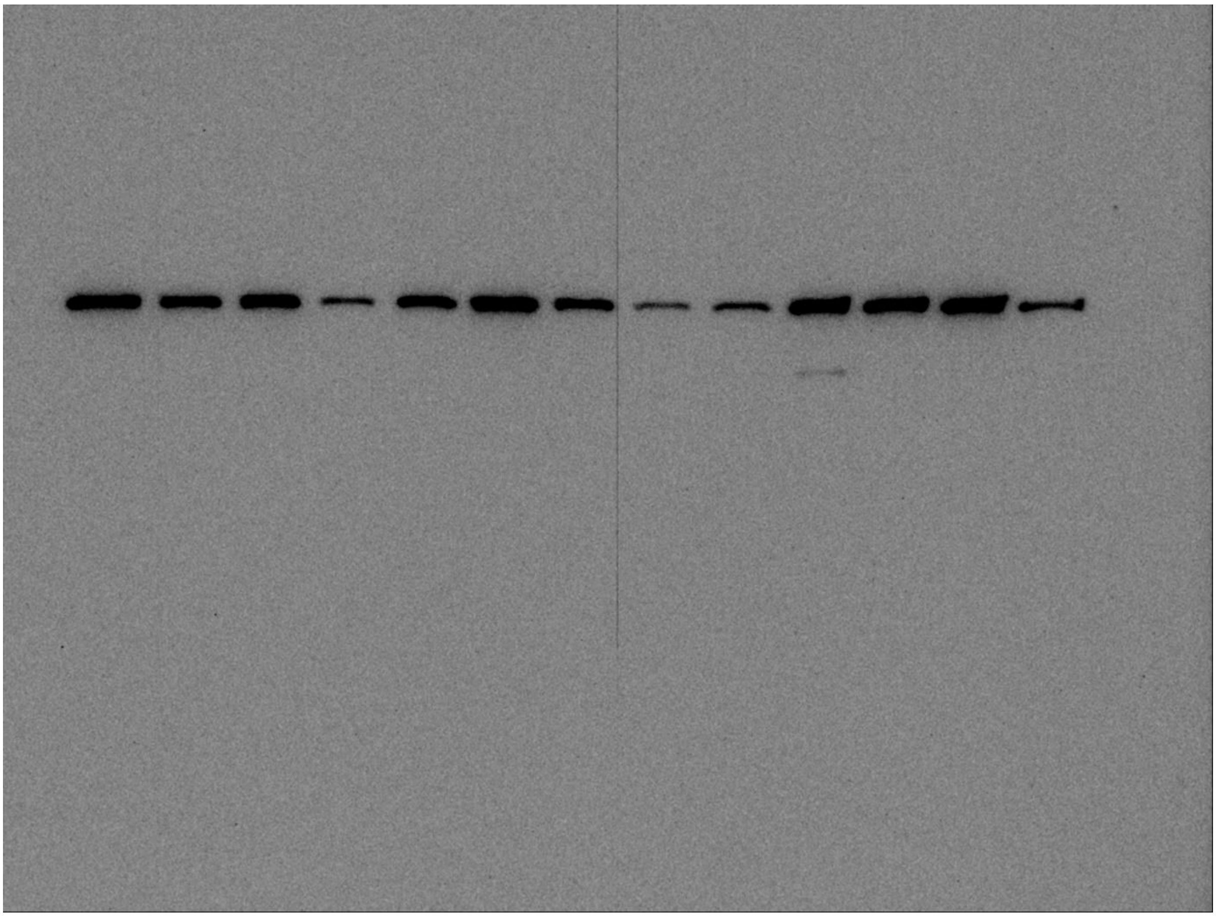

Figure S10. A375 cell line, caspase 7, first band—control, compounds (at 25 and 50 uM) in the order: 17, 6, 37, 1, 15, 16.

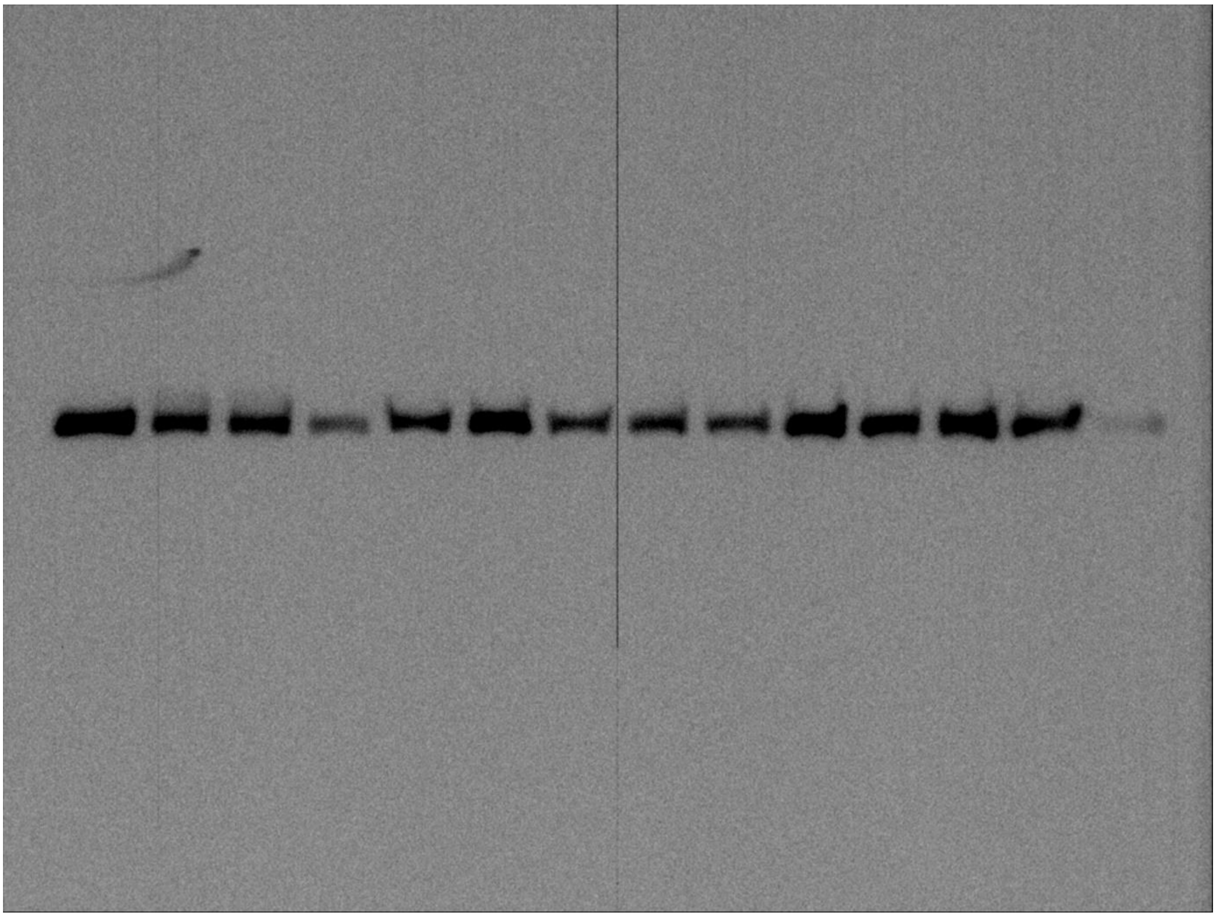

Figure S11. A375 cell line, caspase 3, first band—control, compounds (at 25 and 50 uM) in the order: 17, 6, 37, 1, 15, 16.

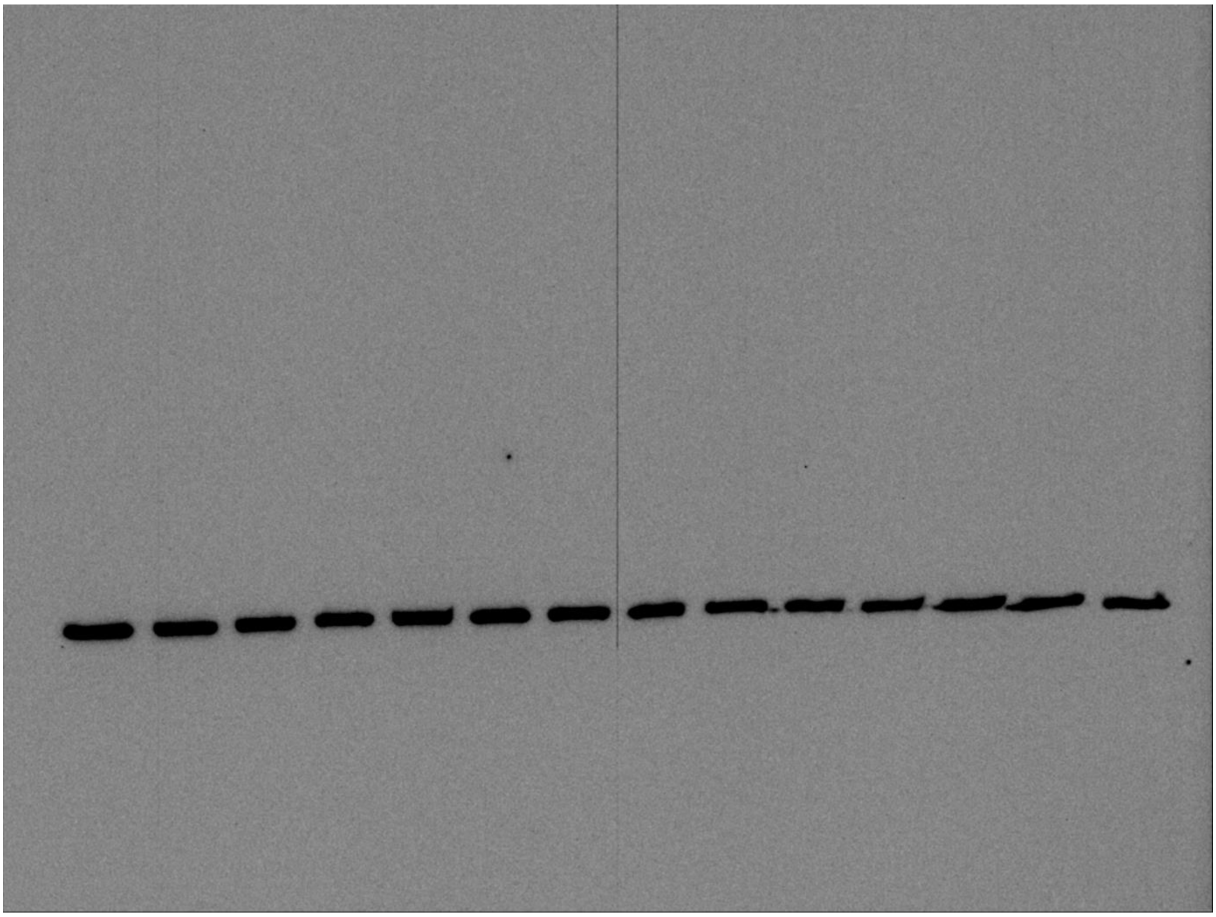

Figure S12. A375 cell line, actin, first band—control, compounds (at 25 and 50 uM) in the order: 17, 6, 37, 1, 15, 16.

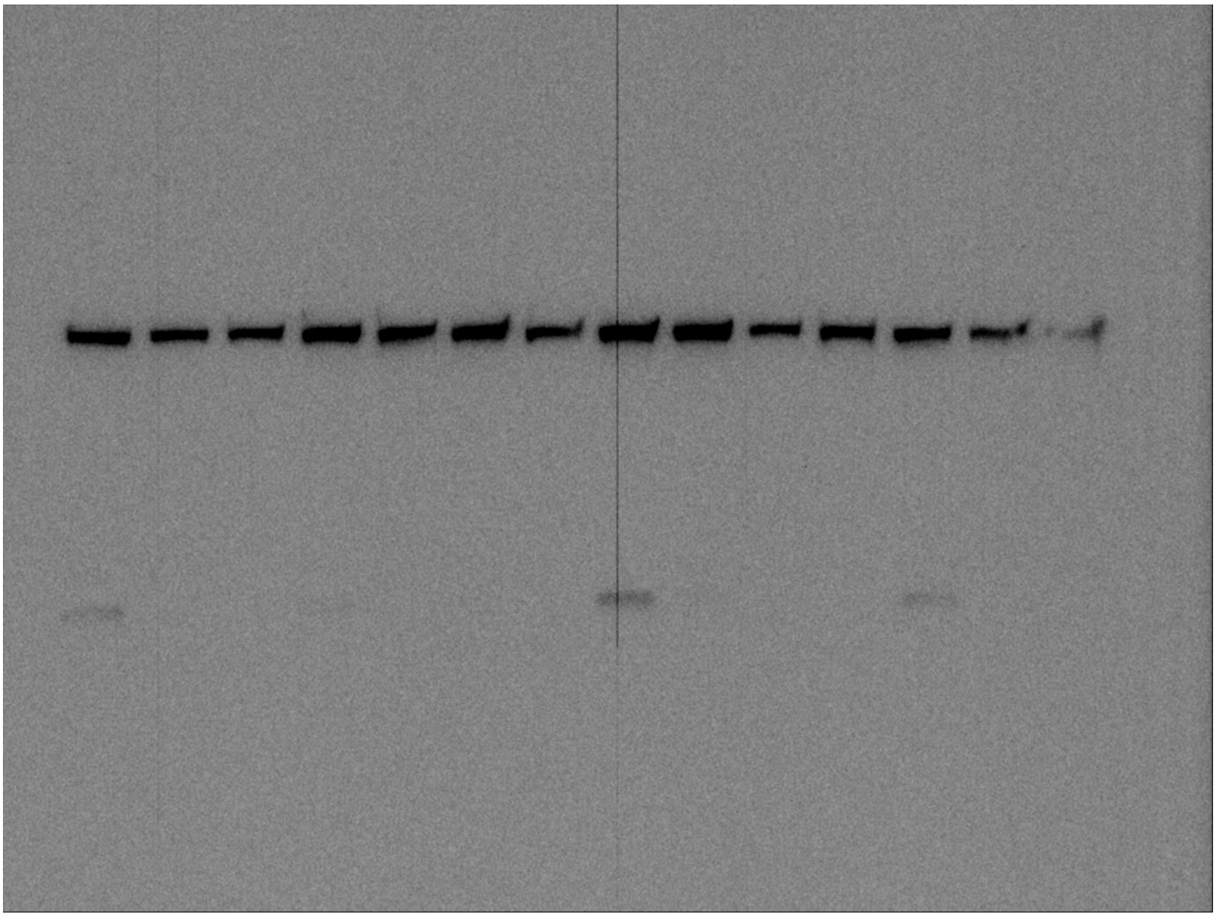

Figure S13. C32 cell line, RSK, first band—control, compounds (at 25 and 50 uM) in the order: 17, 6, 37, 1, 15, 16.

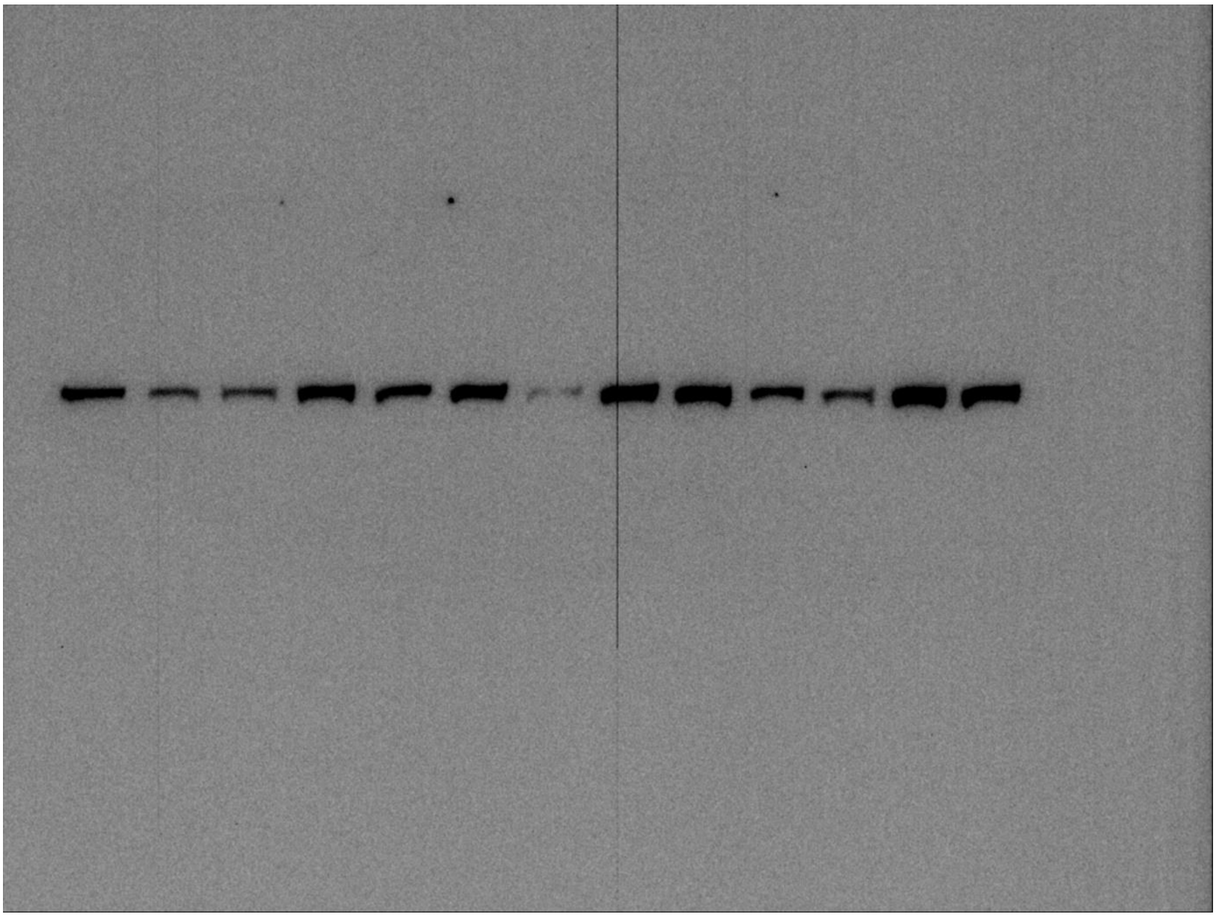

Figure S14. C32 cell line, pRSK, first band—control, compounds (at 25 and 50 uM) in the order: 17, 6, 37, 1, 15, 16.

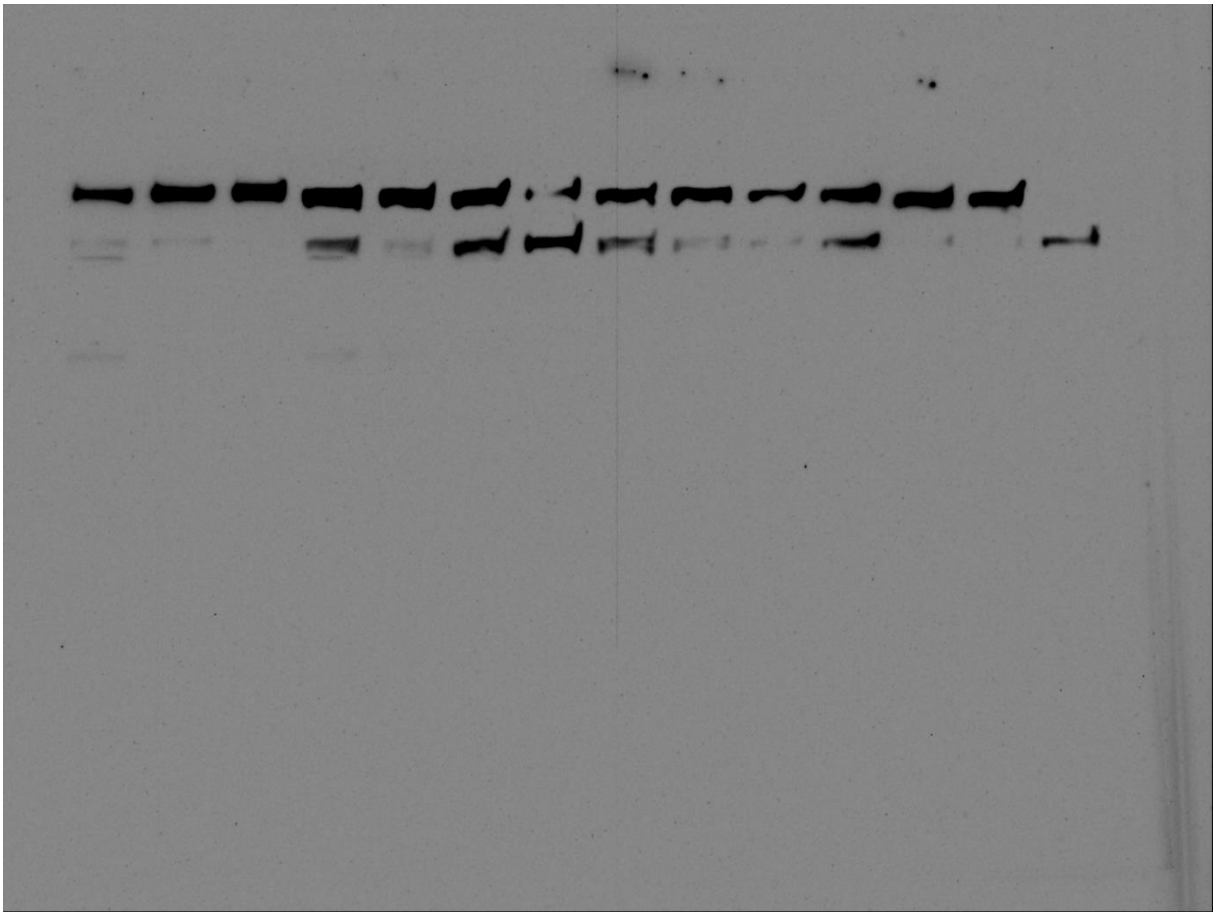

Figure S15. C32 cell line, PARP and cPARP, first band—control, compounds (at 25 and 50 uM) in the order: 17, 6, 37, 1, 15, 16.

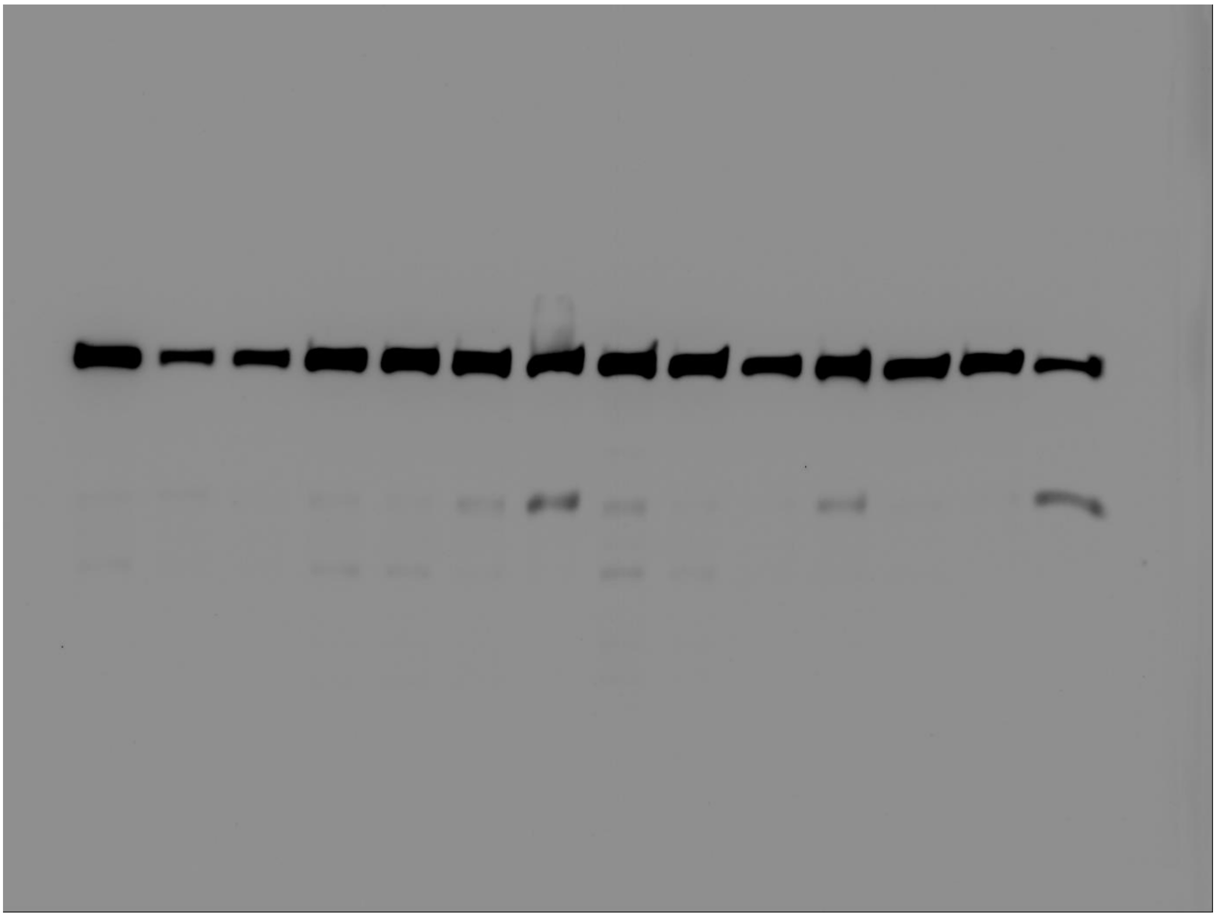

Figure S16. C32 cell line, caspase 9, first band—control, compounds (at 25 and 50 uM) in the order: 17, 6, 37, 1, 15, 16.

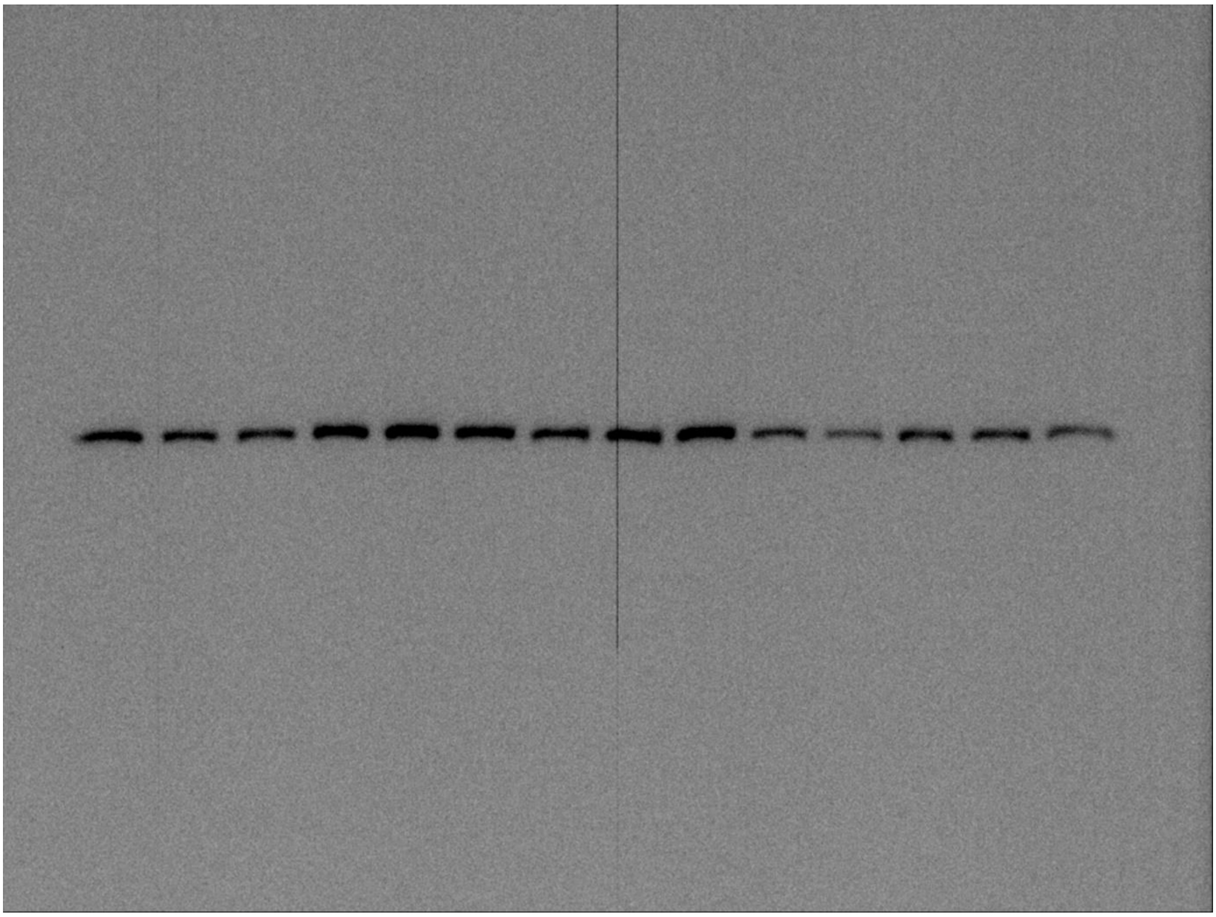

Figure S17. C32 cell line, caspase 7, first band—control, compounds (at 25 and 50 uM) in the order: 17, 6, 37, 1, 15, 16.

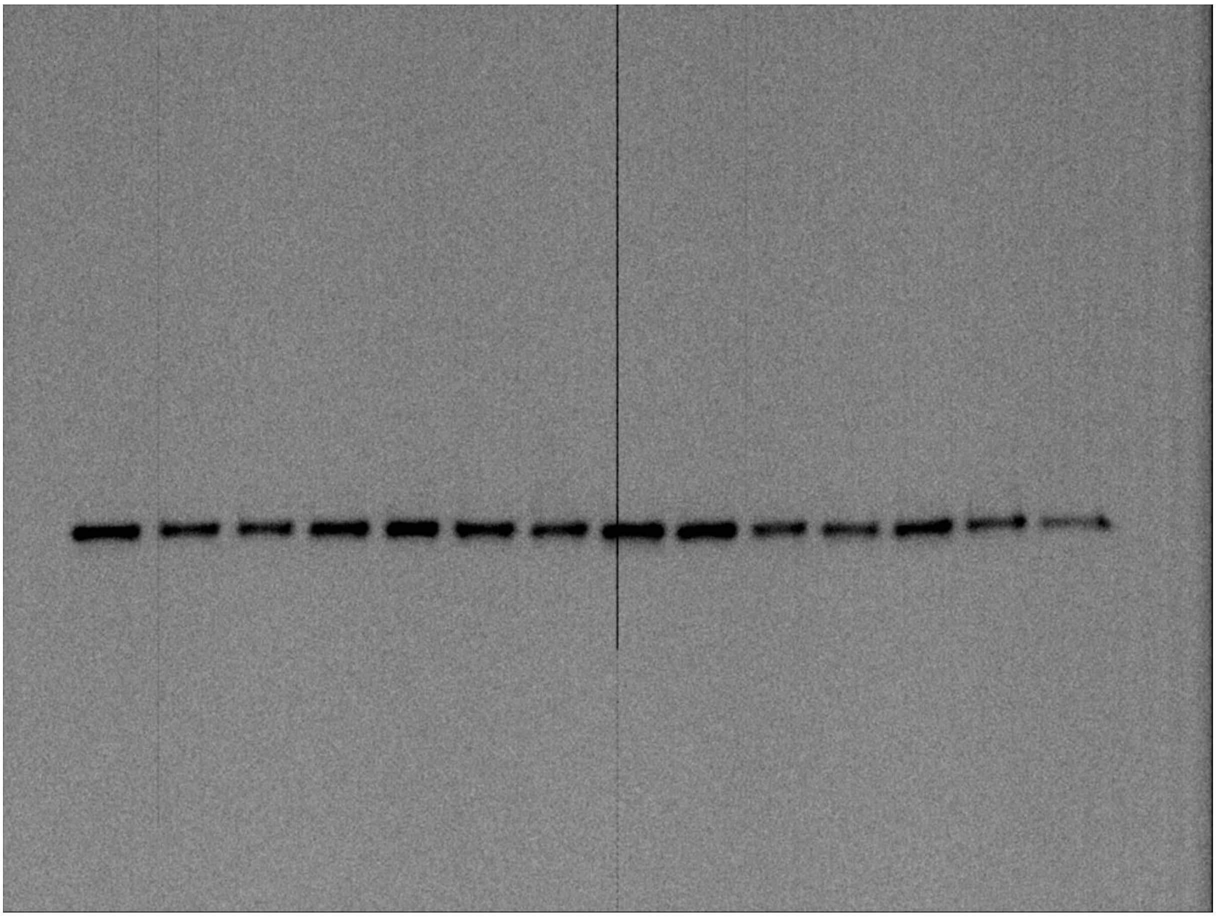

Figure S18. C32 cell line, caspase 3, first band—control, compounds (at 25 and 50 uM) in the order: 17, 6, 37, 1, 15, 16.

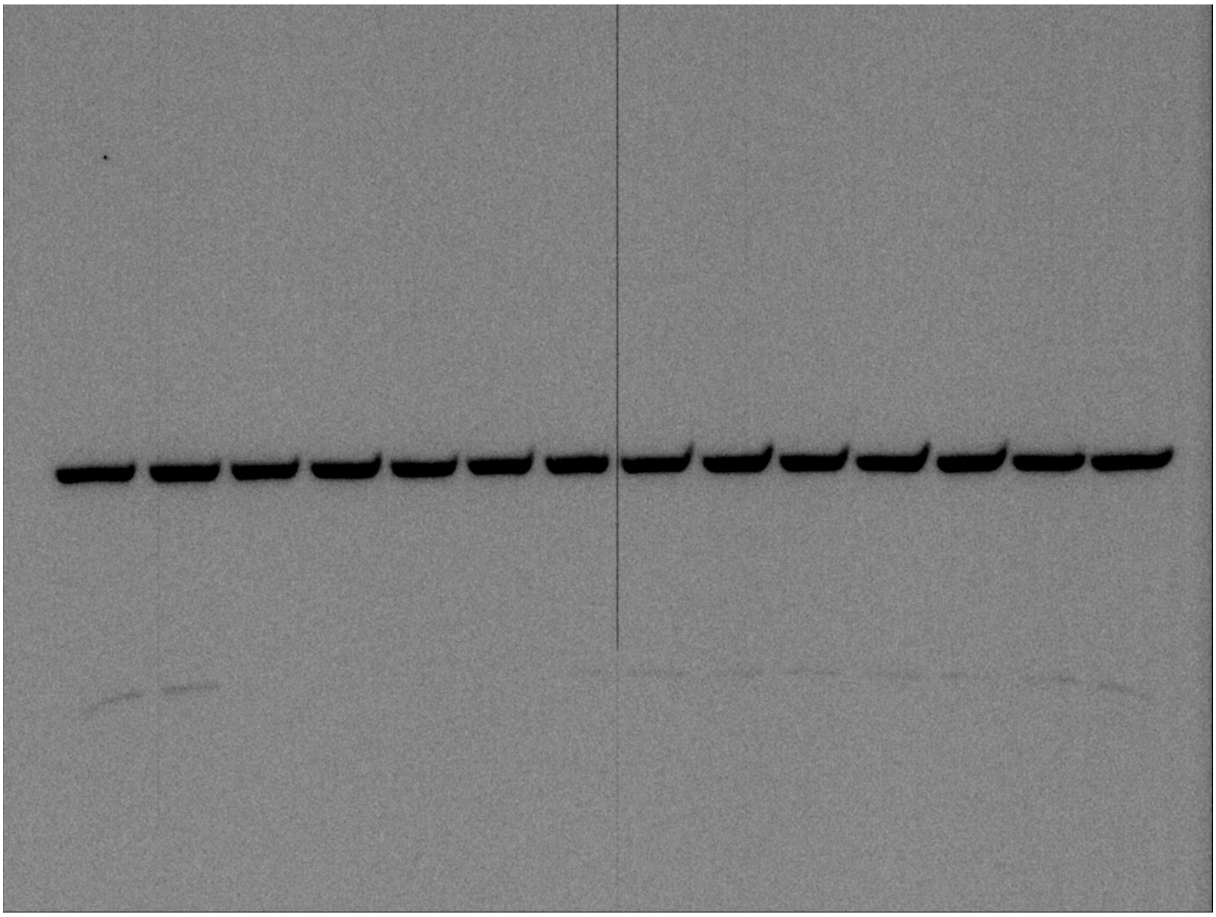

Figure S19. C32 cell line, actin, first band—control, compounds (at 25 and 50 uM) in the order: 17, 6, 37, 1, 15, 16.
